# Endoplasmic Reticulum (ER) Stress Inducible Factor Cysteine-Rich with EGF-Like Domains 2 (Creld2) Is an Important Mediator of BMP9-Regulated Osteogenic Differentiation of Mesenchymal Stem Cells

**DOI:** 10.1371/journal.pone.0073086

**Published:** 2013-09-03

**Authors:** Jiye Zhang, Yaguang Weng, Xing Liu, Jinhua Wang, Wenwen Zhang, Stephanie H. Kim, Hongyu Zhang, Ruidong Li, Yuhan Kong, Xiang Chen, Wei Shui, Ning Wang, Chen Zhao, Ningning Wu, Yunfeng He, Guoxin Nan, Xian Chen, Sheng Wen, Hongmei Zhang, Fang Deng, Lihua Wan, Hue H. Luu, Rex C. Haydon, Lewis L. Shi, Tong-Chuan He, Qiong Shi

**Affiliations:** 1 Ministry of Education Key Laboratory of Diagnostic Medicine and the Affiliated Hospitals of Chongqing Medical University, Chongqing, China; 2 Molecular Oncology Laboratory, Department of Orthopaedic Surgery, The University of Chicago Medical Center, Chicago, Illinois, United States of America; 3 Stem Cell Biology and Therapy Laboratory of the Key Laboratory for Pediatrics co-designated by Chinese Ministry of Education and Chongqing Bureau of Education, The Children’s Hospital of Chongqing Medical University, Chongqing, China; 4 Department of Orthopaedic Surgery, The Affiliated Tangdu Hospital of the Fourth Military Medical University, Xi’an, China; 5 School of Laboratory Medicine and the Affiliated Southwest Hospital of the Third Military Medical University, Chongqing, China; Rush University Medical Center, United States of America

## Abstract

Mesenchymal stem cells (MSCs) are multipotent progenitors that can undergo osteogenic differentiation under proper stimuli. We demonstrated that BMP9 is one of the most osteogenic BMPs. However, the molecular mechanism underlying BMP9-initiated osteogenic signaling in MSCs remains unclear. Through gene expression profiling analysis we identified several candidate mediators of BMP9 osteogenic signaling. Here, we focus on one such signaling mediator and investigate the functional role of cysteine-rich with EGF-like domains 2 (Creld2) in BMP9-initiated osteogenic signaling. Creld2 was originally identified as an ER stress-inducible factor localized in the ER-Golgi apparatus. Our genomewide expression profiling analysis indicates that Creld2 is among the top up-regulated genes in BMP9-stimulated MSCs. We confirm that Creld2 is up-regulated by BMP9 in MSCs. ChIP analysis indicates that Smad1/5/8 directly binds to the Creld2 promoter in a BMP9-dependent fashion. Exogenous expression of Creld2 in MSCs potentiates BMP9-induced early and late osteogenic markers, and matrix mineralization. Conversely, silencing Creld2 expression inhibits BMP9-induced osteogenic differentiation. In vivo stem cell implantation assay reveals that exogenous Creld2 promotes BMP9-induced ectopic bone formation and matrix mineralization, whereas silencing Creld2 expression diminishes BMP9-induced bone formation and matrix mineralization. We further show that Creld2 is localized in ER and the ER stress inducers potentiate BMP9-induced osteogenic differentiation. Our results strongly suggest that Creld2 may be directly regulated by BMP9 and ER stress response may play an important role in regulating osteogenic differentiation.

## Introduction

Mesenchymal stromal/stem cells (MSCs) are multipotent progenitors which can be isolated from numerous tissues, but mostly from bone marrow stromal cells. MSCs can self-renew and differentiate into several lineages, including osteogenic, chondrogenic, and adipogenic lineages [1]–[3]. Osteogenic differentiation is a sequential cascade of events that recapitulates most, if not all, of the skeletal development [4]. As the important members of TGFβ superfamily, BMPs play an important role during development [3], [5], [6] and in osteogenic differentiation [7], [8]. BMPs consist of at least 14 members in humans and rodents [3], [5], [6], [9].

We previously identified BMP9 as one of the most potent BMPs among the 14 types of BMPs in inducing osteogenic differentiation of MSCs both *in vitro* and *in vivo* [6], [10]–[12]. BMP9 (also known as growth differentiation factor 2, or GDF-2) was identified in the developing mouse liver [13] and is one of the least studied BMPs. BMP9 has been shown to play some roles in inducing and maintaining the cholinergic phenotype of embryonic basal forebrain cholinergic neurons [14], inhibiting hepatic glucose production and inducing the expression of key enzymes of lipid metabolism [15], stimulating hepcidin 1 expression [16], and regulating angiogenesis [17]–[24]. Towards understanding the molecular events underlying BMP9-induced osteogenic differentiation, we have demonstrated that TGFβ/BMP type I receptors ALK1 and ALK2 are essential for BMP9-induced osteogenic signaling in MSCs [25]. Through gene expression profiling analysis, we have identified and characterized several early downstream targets of BMP9-induced osteoblast differentiation [6], [12], [26]–[30]. Nonetheless, it remains to be fully elucidated about how BMP9 regulates the cascade events of the osteogenic differentiation.

In this study, we investigate the functional role of cysteine-rich with EGF-like domains 2 (Creld2) in BMP9-initiated osteogenic signaling in MSCs. Although Creld2 is identified as a novel endoplasmic reticulum stress-inducible protein localized in the ER-Golgi apparatus [31], [32], its biological functions are largely undefined. However, it has been reported that ER stress may play an important role in bone formation [33]–[42]. We have recently demonstrated that MAPK signaling pathway is involved in BMP9-induced osteogenic differentiation [43], [44]. Here, our gene expression profiling analysis indicates that Creld2 is among the top up-regulated genes in BMP9-stimulated MSCs. We find that Creld2 is effectively up-regulated in MSCs by BMP9. The ChIP analysis indicates that Smad1/5/8 directly binds to Creld2 promoter in a BMP9-dependent fashion. Exogenous expression of Creld2 in MSCs potentiates BMP9-induced early and late osteogenic markers, as well as matrix mineralization. Conversely, silencing Creld2 expression inhibits BMP9-induced osteogenic differentiation. MSC implantation assay reveals that exogenous Creld2 augments BMP9-induced ectopic bone formation and mature mineralization, whereas silencing Creld2 expression diminishes BMP9-induced bone formation and matrix mineralization. Creld2 is localized on ER in MSCs. Furthermore, Inducers of ER stress potentiate BMP9-osteogenic differentiation. Taken together, our results strongly suggest that Creld2 may be regulated by BMP9 via Smad signaling pathway and that ER stress may play an important role in regulating BMP9-induced osteogenic differentiation of MSCs.

## Materials and Methods

### Cell Culture and Chemicals

HEK-293 and C3H10T1/2 cells were from ATCC (Manassas, VA). HEK293 cells were maintained in complete Dulbecco’s Modified Eagle’s Medium (DMEM) supplemented with 10% fetal calf serum (FCS, Hyclone, Logan, UT, USA), 100 units/ml penicillin, and 100 g/ml streptomycin at 37°C in 5% CO2. whereas C3H10T1/2 cells were in Basal Medium Eagle in Earle’s BSS (BME) supplemented with 10% FCS, 100 units/ml penicillin and 100 g/ml streptomycin at 37°C in 5% CO2 [10], [26], [45], [46]. ER-stress inducers thapsigargin, tunicamycin, and brefeldin were purchased from Sigma-Aldrich (St. Louis, MO). Unless indicated otherwise, all other chemicals were purchased from Sigma-Aldrich or Fisher Scientific (Pittsburgh, PA).

### Microarray Analysis

The microarray analysis was previously described [29]. Briefly, subconfluent C3H10T1/2 cells were maintained in BME medium containing 0.5% FCS and infected with AdBMP9 or AdGFP. Total RNA was isolated at 30 h post infection. The fully characterized RNA samples were used for target preparation and subjected to hybridizations to Affymetrix mouse gene chips 430A. The acquisition and initial quantitation of array images were performed using Affymetrix MAS5.0 with the default parameters (19–21, 31). The acquired microarray raw data were further filtered and normalized to remove noise, whereas retaining true biological information by filtering out the genes with signal intensity in all samples <100 intensity units, and by removing the genes that received an “absent” call for all hybridizations. The clustering analysis was carried out by using the DNA-Chip Analyzer (dChip) software (51). Thresholds for selecting significant genes were set at a relative difference>2-fold, an absolute difference >100 signal intensity units, and a statistical difference at *p*<0.05. The top 20 BMP9 up-regulated genes are listed in Table S1. The GEO accession number for the microarray dataset is GSE48882.

### Construction of Recombinant Adenoviruses Expressing BMP9, Creld2, and simCreld2

Recombinant adenoviruses were generated using AdEasy technology as described [10], [11], [47]–[49]. The coding regions of human BMP9 and mouse Crelds were PCR amplified and cloned into an adenoviral shuttle vector and subsequently used to generate recombinant adenoviruses in HEK-293 cells. The siRNA target sites against mouse Creld2 coding region were selected by using Dharmacon’s *siDESIGN* program (Table S2), and the siRNA oligonucleotide pairs were cloned into the pSES adenoviral shuttle vector [50] to generate recombinant adenoviruses. The resultant adenoviruses were designated as AdBMP9, AdR-Creld2, or AdR-simCreld2. AdBMP9 also expresses GFP, whereas AdR-Creld2 and AdR-simCreld2 express RFP as a marker for monitoring infection efficiency. Analogous adenovirus expressing only monomeric RFP (AdRFP) or GFP (AdGFP) were used as controls [27]–[29], [46], [47], [49], [51].

### Quantitative (qPCR) and Semi-Quantitative RT-PCR (sqPCR) Analysis

Total RNA was isolated using TRIZOL Reagents (Invitrogen) and used to generate cDNA templates by RT reaction with hexamer and M-MuLV Reverse Transcriptase (New England Biolabs, Ipswich, MA). The first strand cDNA products were further diluted 5- to 10-fold and used as PCR templates. The SYBR Green-based qPCR and/or sqPCR were carried out as described [52]–[56]. PCR primers (Table S2) were designed by using the Primer3 program and used to amplify the genes of interest (approximately 150–180 bp). A touchdown cycling program was as follows: 94°C for 2 min for 1 cycle; 92°C for 20 s, 68°C for 30 s, and 72°C for 12 cycles decreasing 1°C per cycle; and then at 92°C for 20 s, 57°C for 30 s, and 72°C for 20 s for 20–25 cycles, depending on the abundance of target genes. PCR products were resolved on 1.5% agarose gels. All samples were normalized by the expression level of GAPDH.

### Chromatin Immunoprecipitation (ChIP) Analysis

Subconfluent C3H10T1/2 cells were infected with AdGFP or AdBMP9. At 30 h after infection, cells were cross-linked and subjected to ChIP analysis as previously described [29], [46], [51]. Smad1/5/8 antibody (Santa Cruz Biotechnology) or control IgG was used to pull down the protein-DNA complexes. The presence of *Creld2* promoter sequence was detected by using two pairs of primers corresponding to mouse *Creld2* promoter region.

### Alkaline Phosphatase (ALP) Activity Assays

ALP activity was assessed by a modified Great Escape SEAP Chemiluminescence assay (BD Clontech, Mountain View, CA) and/or histochemical staining assay (using a mixture of 0.1 mg/ml napthol AS-MX phosphate and 0.6 mg/ml Fast Blue BB salt) as described [10], [11], [27]–[29], [45], [46], [48], [53], [57]. For the chemilluminescence assays, each assay condition was performed in triplicate. The results were repeated in at least three independent experiments.

### Alizarin Red S Staining

C3H10T1/2 cells were seeded in 24-well cell culture plates and infected with adenoviruses AdBMP-9 and AdR-simCreld2 or AdCreld2. The cells were cultured in the presence of ascorbic acid (50 µg/mL) and β-glycerophosphate (10 mM) for 10–14 days. Mineralized matrix nodules were stained for calcium precipitation by means of Alizarin Red S staining as described previously [10], [11], [27]–[29], [45], [46], [48]. Cells were fixed with 0.05% (v/v) glutaraldehyde at room temperature for 10 min. After being washed with distilled water, fixed cells were incubated with 0.4% Alizarin Red S for 5 min, followed by extensive washing with distilled water. The staining of calcium mineral deposits was recorded under a bright field microscope.

### Stem Cell Implantation and µCT Analysis

The use and care of the animals in this study were approved by The University of Chicago Institutional Animal Care and Use Committee (IACUC) (Protocol #71108). Briefly, MSCs were infected with AdBMP9/AdRFP, AdBMP9/AdR-Creld2, or AdBMP9/AdR-simCreld2. At 16 h post infection, cells were harvested and resuspended in PBS for subcutaneous injection (5×10^6^/injection) into the flanks of athymic nude (nu/nu) mice (5 animals/group, 4–6 week-old, female, Harlan Sprague-Dawley). At 4 wk post implantation, animals were sacrificed. Implantation sites were retrieved for µCT analysis, histologic evaluation, and other stains. All specimens were imaged using the µCT component of the GE Triumph (GE Healthcare, Piscataway, NJ, USA) trimodality preclinical imaging system. All image data analysis was performed using Amira 5.3 (Visage Imaging, Inc., San Diego, CA, USA); and 3D volumetric data and bone mean density heat maps were obtained as previously described [25], [53], [57].

### Hematoxylin & Eosin, Trichrome, and Alcian Blue Staining

Retrieved tissues were fixed, decalcified in 10% buffered formalin, and embedded in paraffin. Serial sections of the embedded specimens were stained with hematoxylin and eosin (H & E). Masson’s Trichome and Alcian Blue stains were carried out as previously described [11], [25], [28], [29], [45], [46], [48], [53], [57].

### Construction of the cGFP-Creld2 Fusion Protein

The coding region (without the stop codon) of mouse Creld2 was PCR amplified and subcloned in frame at the N-terminus of copGFP in homemade retroviral expression vector, designated as Creld2-cGFP. The cloning junctions and PCR amplified fragment were verified by DNA sequencing. The parental copGFP vector was used as a control. The vector DNA was purified by using the Wizard miniprep kit (Promega) and used for transfection of iMEFs in culture.

### Statistical Analysis

All quantitative experiments were performed in triplicate and/or repeated three times. Data were expressed as mean ±SD. Statistical significances between treatment *vs.* control treatment were determined by one-way analysis of variance and the Student’s *t* test. A value of *p*<0.05 was considered statistically significant.

## Results

### Creld2 Is One of the Significantly Up-Regulated Genes in BMP9-Stimulated MSCs

**Figure 1:**
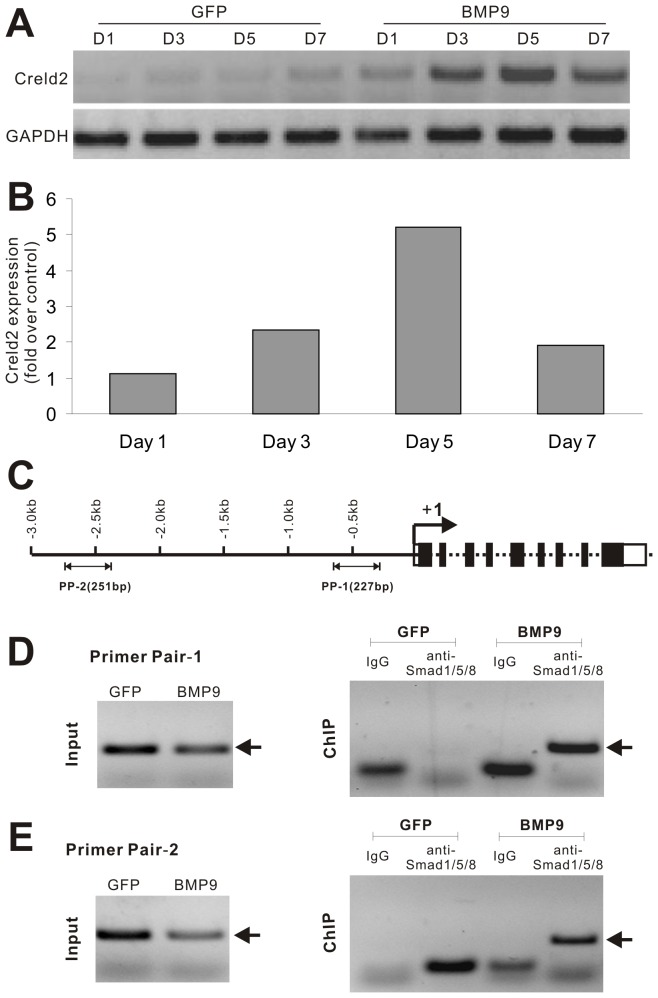
BMP9 regulates Creld2 expression via Smad signaling pathway in MSCs. (A) Time course expression of Creld2 upon BMP9 stimulation using sqPCR. Subconfluent C3H10T1/2 cells were cultured in 1% FBS DMEM and infected with AdBMP9 or AdGFP. Total RNA was collected at the indicated time points and subjected to semi-quantitative RT-PCR analysis. All samples were normalized for GAPDH expression. (B) qPCR analysis of BMP9-induced Creld2 expression. RT-PCR samples prepared from (A) were used for SYBR Green-based qPCR analysis. The expression level of GAPDH was used as an internal control. (C) A schematic presentation of the 3.0 kb promoter region of mouse Creld2. (D) & (E) ChIP analysis of the mouse Creld2 promoter.C3H10T1/2 cells were infected with AdBMP9 or AdGFP for 36 h followed by formaldehyde cross-linking. The cross-linked cells were lysed and subjected to sonication and immunoprecipitation using anti-Smad1/5/8 (Santa Cruz biotechnology Inc., Cat# sc-6031-R) or control (rabbit) IgG. The recovered chromatin DNA fragments were used for PCR amplifications with two pairs of primers specific for the mouse Creld2 promoter. The expected PCR products are indicated by arrows.

We previously demonstrated that BMP9 is one of the most osteogenic factors for inducing osteoblastic differentiation in MSCs [3], [6], [10]–[12]. Through gene expression profiling analysis, we have identified several downstream targets that may play important roles in mediating BMP9-induced osteogenic signaling [12], [27]–[30]. Nonetheless, the exact molecular mechanisms underlying BMP9 functions in MSCs remain to be fully elucidated. Here, we investigated if Creld2 plays any role in BMP9 osteogenic signaling in MSCs, as Creld2 was identified as one of the top up-regulated genes in MSCs upon BMP9 stimulation (Table S1) [29]. Using sqPCR analysis, we found that Creld2 was up-regulated in MSC line C3H10T1/2 cells at 24 h, peaked at day 5 post BMP9 transduction (Figs. 1A and 1B). Similar results were obtained in other MSCs, including primary bone marrow stromal cells and iMEFs [58] (data not shown). These results confirm the microarray analysis data and suggest that Creld2 may function as a downstream target of BMP9 signaling.

### Creld2 is a Direct Target of BMP9/Smad Signaling Pathway

We next analyzed if Creld2 is a direct target of BMP9 signaling. ChIP analysis would allow us to determine whether Creld2 promoter can interact with the BMP-specific Smad1/5/8. We conducted ChIP analysis using Smad1/5/8 antibody or isotype IgG to pull down genomic DNA fragment from MSCs transduced with BMP9 or GFP. Two pairs, PP-1 and PP-2, of mouse Creld2 promoter-specific primers located within the proximal 3 kb region were chosen (Figure 1C). The PP-1 is located about 500 bp upstream of exon-1 and was shown to pull-down the expected product that bound to Smad1/5/8 upon BMP9 stimulation (Figure 1D). Likewise, the PP-2 is located about 2.5 kb upstream and also pulled down by Smad1/5/8 antibody in a BMP9-dependent fashion (Figure 1E). These results suggest that Creld2 expression may be directly regulated by BMP9 through BMP-specific R-Smad1/5/8.

### Exogenous Expression of Creld2 Potentiates BMP9-Induced Early Osteogenic Marker ALP in MSCs, which can be Blunted by Silencing Creld2 Expression

**Figure 2:**
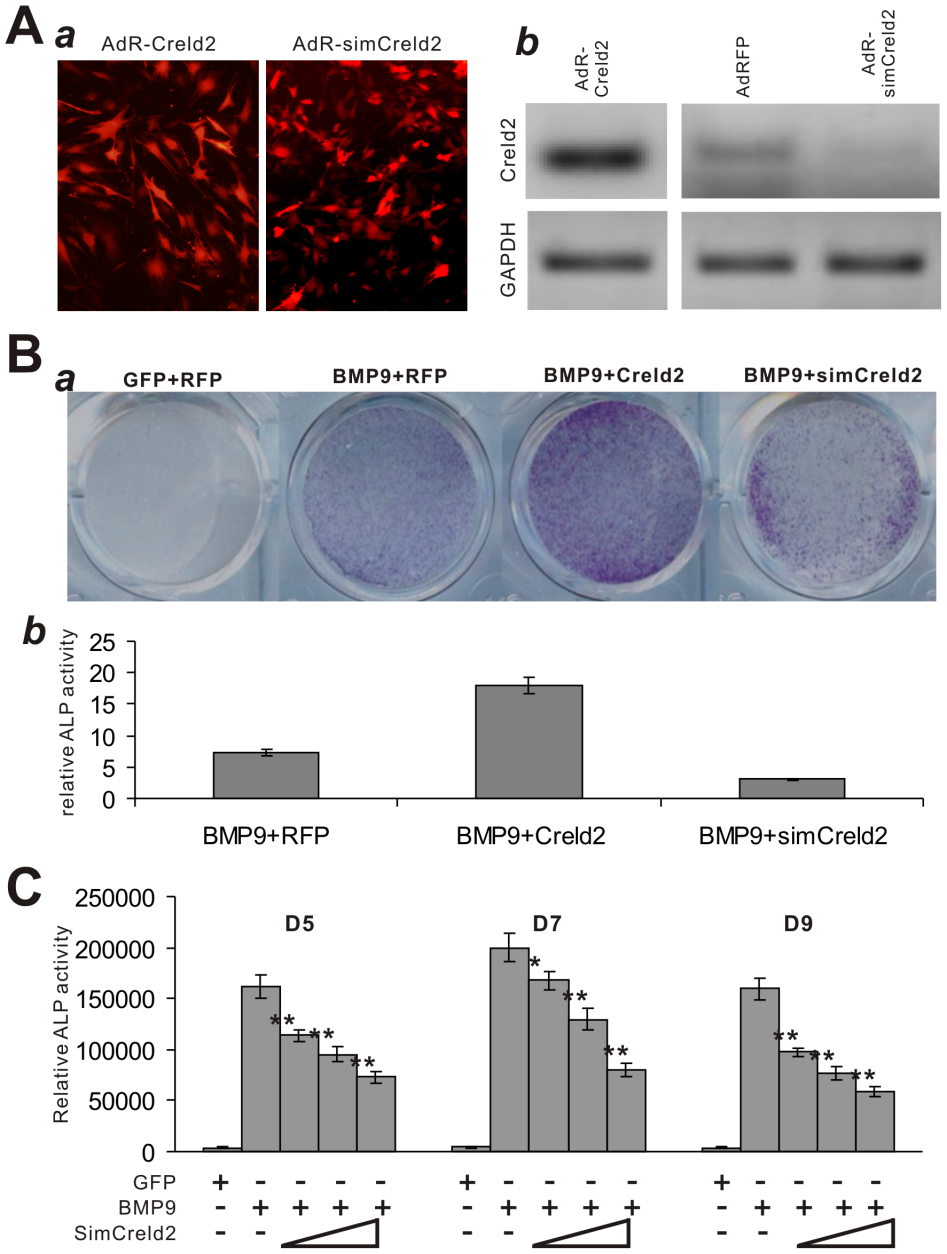
Exogenous Creld2 expression potentiates BMP9-induced early and late osteogenic markers in MSCs, which is diminished by silencing Creld2 expression. (A) Construction of the recombinant adenovirus expressing Creld2 or silencing Creld2 expression in MSCs. The recombinant adenovirus AdR-Creld2 and AdR-simCreld2 were shown to effectively transduce MSCs, such as C3H10T1/2 (*a*). For adenovirus-mediated exogenous Creld2 expression or silencing Creld2 expression, C3H10T1/2 cells were infected with Ad-RFP, AdR-Creld2, or AdR-simCreld2. Total RNA was isolated at 72 h post infection and subjected to sqPCR using primer pairs specific for mouse Creld2 (*b*). (B) Creld2 potentiates BMP9-induced ALP activity in MSCs. Subconfluent C3H10T1/2 cells were co-infected with AdBMP9, AdR-Creld2, AdR-simCreld2, and/or AdGFP. ALP activity was measured at day 7 by histochemical staining (*a*) and chemiluminescent assays (*b*). (C) Silencing Creld2 expression inhibits BMP9-induced ALP activity in MSCs. C3H10T1/2 cells were infected with AdBMP9 or AdGFP and escalating titers of AdR-simCrelds virus. At the indicated time points post infection, the ALP activity was assessed. “**”, *p<0.001*; “*”, *p<0.05*. Each assay condition was done in triplicate and/or carried out at least in three independent experiments. Representative results are shown.

If Creld2 is an important target of BMP9-mediated osteogenic signaling, we hypothesized that exogenous expression of Creld2 could enhance BMP9-induced osteogenic differentiation of MSCs, whereas silencing Creld2 expression would inhibit BMP9-induced osteogenic signaling. To effectively introduce exogenous Creld2 or silence Creld2 expression in MSCs, we constructed recombinant adenovirus expressing mouse Creld2 (AdR-Creld2) or siRNAs targeting mouse Creld2 coding region (AdR-simCreld2) [50]. We demonstrated that both adenoviral vectors can transduce MSCs with high efficiency as both co-express RFP marker (Figure 2A, panel a). Adenovirus-mediated transgene expression and knockdown of endogenous Creld2 were verified by semi-quantitative RT-PCR (Figure 2A, panel b).

While exogenous Creld2 expression alone did not exert any significant effect on osteogenic early marker alkaline phosphatase (ALP) activity (data not shown), Creld2 was shown to exhibit a profound synergistic effect on BMP9-induced ALP activity in C3H10T1/2 MSCs (Figure 2B, panel a). Quantitatively, Creld2-mediated synergistic effect on ALP activity in BMP9-transduced C3H10T1/2 cells was increased by 140% on day 7 (Figure 2B, panel b). Conversely, BMP9-induced ALP activity was significantly inhibited in MSCs by AdR-simCreld2 to 40% of the BMP9 control’s on day 7 (Figure 2B, panel b). In fact, BMP9-induced ALP activity could be effectively inhibited by AdR-simCreld2 in a dose-dependent fashion (Figure 2C). These results suggest that Creld2 may be an important mediator of BMP9 osteogenic signaling.

### Creld2 is Essential for BMP9-Induced Terminal Osteogenic Differentiation and Matrix Mineralization of MSCs in vitro

**Figure 3:**
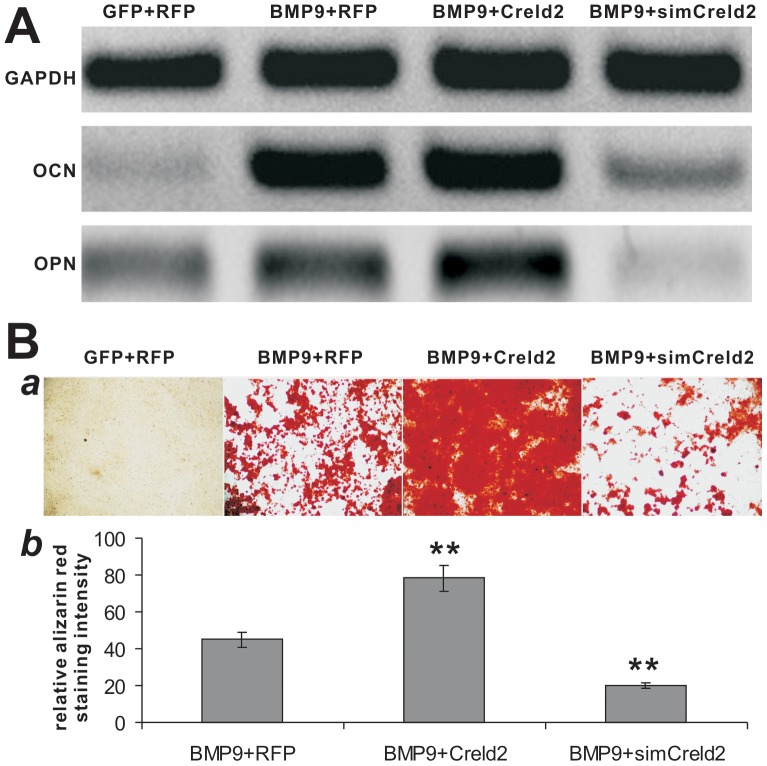
Knockdown of Creld2 expression diminishes BMP9-induced late osteogenic markers and matrix mineralization of MSCs *in vitro,* which are augmented by Creld2 overexpression. (A) Silencing Creld2 blunts BMP9-induced expression of OPN and OCN. Subconfluent C3H10T1/2 cells were co-infected with the indicated adenoviral vectors. At 7 days post post infection, total RNA was isolated for sqPCR analysis using primers specific for mouse OPN, OCN, and GAPDH (as a control). (B) Silencing Creld2 inhibits BMP9-induced matrix mineralization. C3H10T1/2 cells were co-infected with the indicated adenoviral vectors. Alizarin Red S staining was conducted at 10 days after infection (*a*). The staining was further quantitatively analyzed (*b*). Each assay condition was done in triplicate and/or carried out at least in three independent experiments. “**”, *p<0.001*; Representative results are shown.

We further analyzed the effect of Creld2 on the expression of late osteogenic markers osteopotin (OPN) and osteocalcin (OCN) in BMP9-stimulated MSCs. While exogenous Creld2 expression slightly augmented the expression of both OPN and OCN, silencing Creld2 expression effectively reduced the expression levels of OPN and OCN (Figure 3A). Furthermore, we found Creld2 expression significantly increased the BMP9-induced matrix mineralization as assessed by alizarin red staining, where silencing Creld2 led to a decrease in alizarin red staining (Figure 3B, panel a). Quantitative analysis of the alizarin red staining indicates that the BMP9-induced matrix mineralization was significantly enhanced by Creld2 overexpression while inhibited by silencing Creld2 (p<0.01) (Figure 3B, panel b). Taken these in vitro results together, Creld2 has been shown to potentiate BMP9-induced osteoblastic commitment and terminal differentiation of MSCs in vitro.

### Creld2 Plays an Important Role in BMP9-induced Terminal Differentiation and Ectopic Bone Formation in vivo

**Figure 4:**
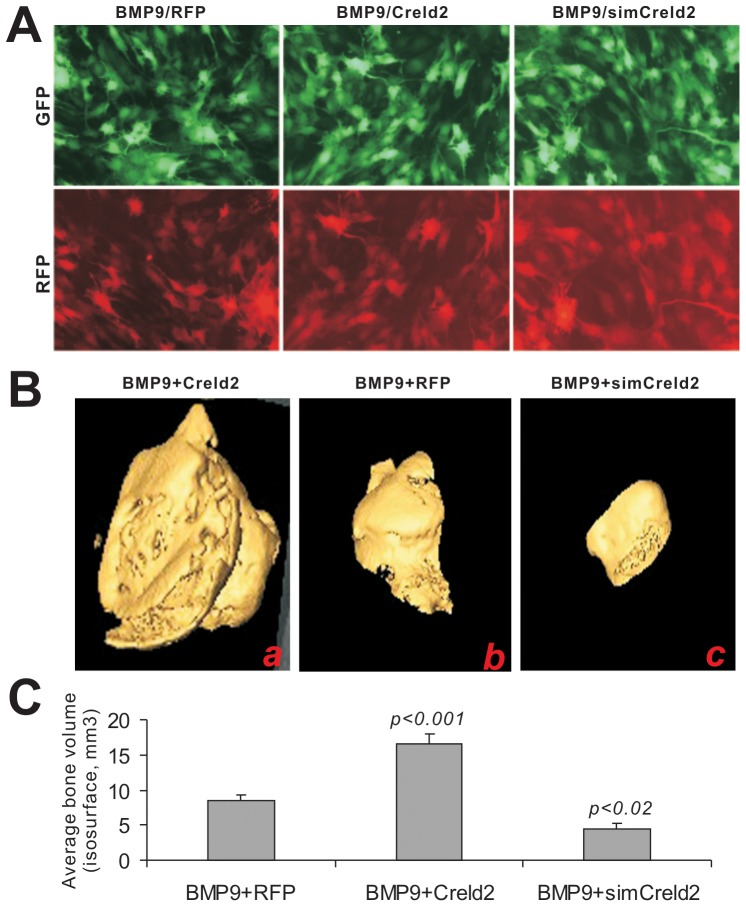
Exogenous Creld2 enhances, while silencing Creld2 inhibits BMP9-induced ectopic bone formation. C3H10T1/2 cells were co-transduced with BMP9, RFP, Creld2 and/or simCreld2 adenoviruses for 16 h (A) and collected for subcutaneous injections into the flanks of athymic nude mice. Bony masses were collected at 4 weeks and subjected to microCT analysis to determine the 3-dimensional iso-surface (B) and the mean mineralization density (C). No masses were detected in the subcutaneous injections, in which the implanted cells were transduced with RFP, Creld2, or simCreld2 without BMP9. The p-values were calculated by comparing the results from BMP9/Creld2 or BMP9/simCreld2 group with that the BMP9/RFP’s. Representative images are shown.

While the above in vitro studies established that Creld2 may play an important role in BMP9-mediated osteogenic signaling, it was essential to determine if Creld2 plays such a role in vivo. We chose to use our previously established stem implantation assay [25], [30], [53], [57], [58]. MSCs were first transduced with BMP9 and Creld2, simCreld2, or RFP for 16 h (Figure 4A). The cells were collected and injected subcutaneously into the flanks of athymic nude mice for 4 weeks. MSCs transduced with RFP, Creld2 or simCreld2 alone did not form any detectable masses (data not shown). We found that Creld2 over-expression significantly augmented BMP9-induced bony mass formation whereas simCreld2 inhibited BMP9-induced bone formation (Figure 4B). The gross size differences were quantitatively assessed by iso-surface 3-dimensional analysis of the µCT imaging data (Figure 4C). Conversely, silencing Creld2 expression significantly decreased the average bone volume (Figures 4B and 4C).

**Figure 5:**
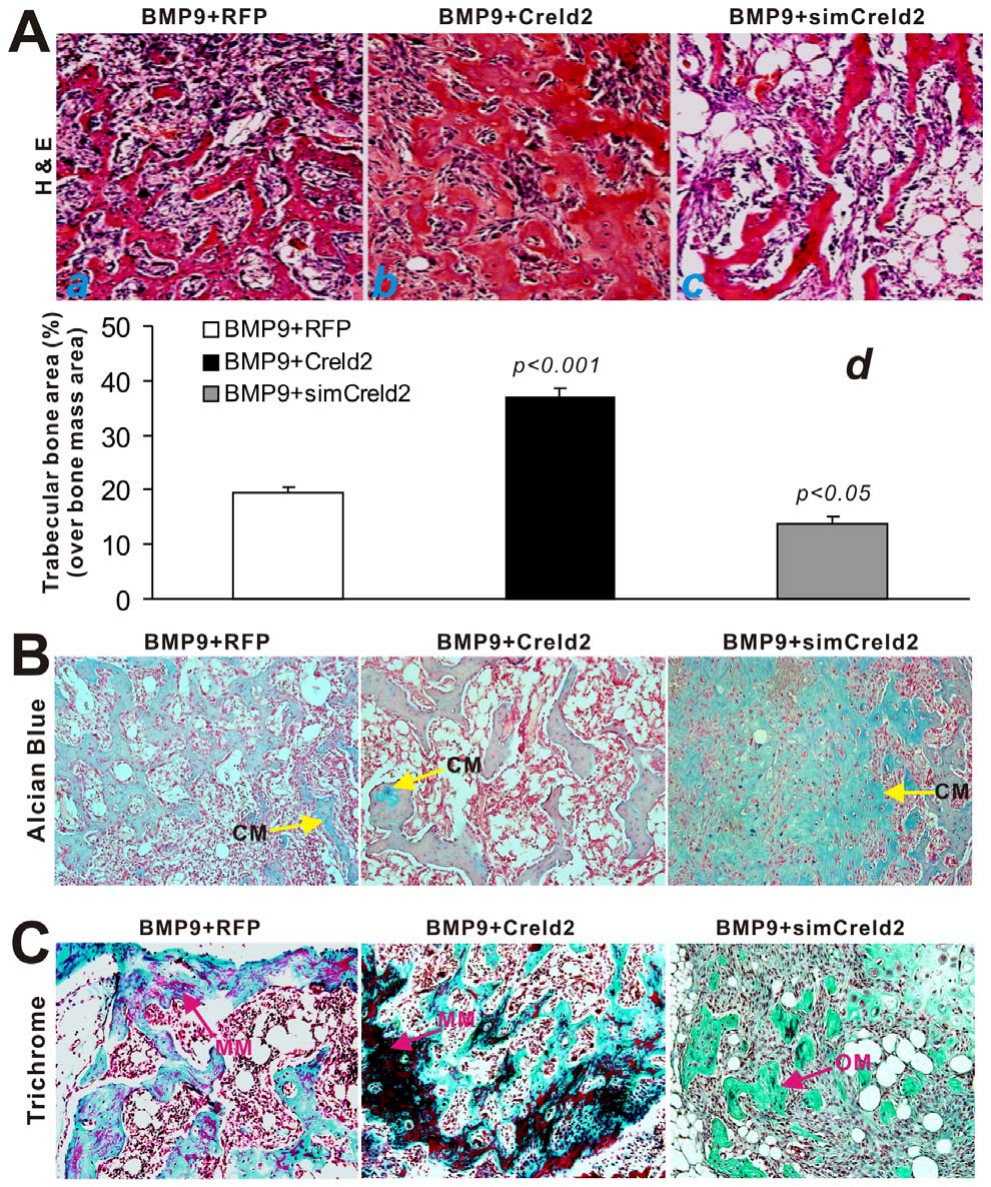
Creld2 potentiates BMP9-induced terminal osteogenic differentiation and matrix mineralization. The retrieved samples were fixed, decalcified, paraffin-embedded, and subjected to histologic analysis. (A) H & E staining. Sections from the retrieved samples of BMP9/RFP (*a*), BMP9/Creld2 (*b*), and BMP9/simCreld2 (*c*) were subjected to H & E staining. The trabecular structures, including % of trabecular area of the total area (*d*), were quantitatively analyzed using the ImageJ software. The p-values were calculated by comparing the results from BMP9/Creld2 or BMP9/simCreld2 group with that the BMP9/RFP’s. (B) Alcian blue staining. CM, cartilage matrix. (C) Masson’s Trichrome staining. MM, mineralized matrix; OM, osteoid matrix. Magnification, 200×. Representative results are shown.

The retrieved samples were further subjected to histologic analysis and other special staining. H & E staining revealed that Creld2 significantly enhanced BMP9-induced bone formation and mineralization and that silencing Creld2 inhibited BMP9-induced osteogenesis (Figure 5A panels a vs. b). Quantitative analysis indicates that co-expression of BMP9 and Creld2 significantly increased the average thickness of trabeculae and the percentage of trabecular area over total area (Fig, 5A, panel d), whereas knocking down Creld2 expression exhibited an inhibitory effect (Figure 5A, panels a vs. c). The alcian blue staining revealed that silencing Creld2 expression led to the accumulation of cartilaginous matrix in BMP9-transduced MSCs, compared with that of the BMP9 alone group, whereas the BMP9+Creld2 group exhibited a slight decrease in alcian blue staining (Figure 5B). These results suggest that Creld2 may facilitate BMP9-induced terminal osteogenic differentiation. This notion was further confirmed by Masson’s trichrome staining assays. We found that, in the presence of exogenous Creld2, BMP9 induced robust and highly mature bone matrix mineralization, while BMP9 failed to induce the formation of mature bone matrix from MSCs when Creld2 expression was silenced (Figure 5C). These in vivo findings are supported by the in vitro studies. Collectively, our results strongly indicate that Creld2 is an important mediator of BMP9-induced terminal osteogenic differentiation, and that exogenous Creld2 expression augments BMP9-induced osteogenic differentiation and hence produces more mature bone.

### ER-stress Inducers Enhance BMP9-Induced Osteogenic Differentiation of MSCs

**Figure 6:**
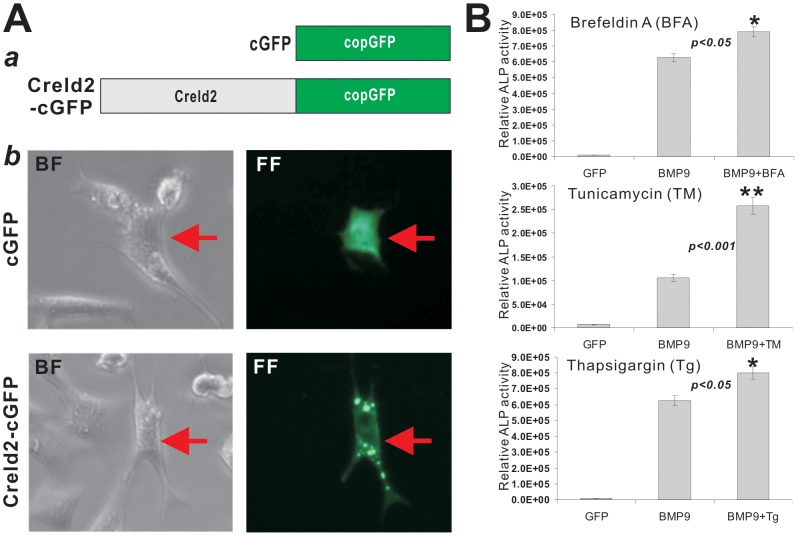
ER stress inducers potentiate BMP9-induced osteogenic differentiation. (A) ER localization of Creld2 in MSCs. (a) Schematic representation of copGFP-tagged Creld2. The coding region (without stop codon) of mouse Creld2 was PCR amplified and cloned in frame at the N-terminus of copGFP into an expression vector, resultant Creld2-cGFP. The parental copGFP (cGFP) was used as a control. (b) Tracking the ER location of Creld2-cGFP in MSCs. Subconfluent iMEFs were transfected with Creld2-cGFP or cGFP using Lipofectamine. Fluorescence signal and bright field images were recorded at 24 h post transfection. Transfected cells are indicated by arrows. Representative results are shown. (B) Inducers of ER stress potentiate BMP9-induced osteogenic differentiation in MSCs. Subconfluent iMEFs were infected with AdBMP9 or AdGFP in the presence of Brefeldin (0.2 µg/ml), Tunicamycin (0.005 µg/ml), Thapsigargin (0.0001 µM), or vehicle only. After 5 days, ALP activity was determined. Each assay condition was done in triplicate. “*”, *p<0.05*; “**”, *p<0.001*.

The Creld2’s biological functions remain unclear, except that it was reported that Creld2 is located on ER and induced during ER stress [31], [32]. To determine the cellular location of Creld2 in MSCs, we fused the Creld2 with copGFP (Figure 6A, panel a) and confirmed that Creld2-cGFP is located in the ER structure of MSC cells (Figure 6A, panel b). We further examined the effect of three commonly-used ER-stress inducers on BMP9-induced osteogenic differentiation. All three inducers (thapsigargin, tunicamycin, and brefeldin) were shown to enhance BMP9-induced ALP activity although tunicamycin potentiated ALP activity most pronouncedly (Figure 6B). The inducers alone did not induce any detectable ALP activity in iMEFs (data not shown). These above results suggest that the induction of ER stress may play an important role in BMP9-induced osteogenic differentiation, although the detailed mechanism remains to be elucidated.

## Discussion

We have demonstrated that BMP9 is the most potent osteogenic BMP both *in vitro* and *in vivo* [6], [10]–[12]. However, the biological functions of BMP9 in bone and musculoskeletal system remain to be fully investigated. In this study, we investigate the functional role of cysteine-rich with EGF-like domains 2 (Creld2) in BMP9-initiated osteogenic signaling in MSCs. We demonstrate that Creld2 is induced in MSCs upon BMP9 stimulation with a peak induction at day 5. ChIP analysis reveals that Smad1/5/8 directly binds to Creld2 promoter region in a BMP9-dependent fashion. Exogenous expression of Creld2 in MSCs potentiates BMP9-induced early and late osteogenic markers, as well as matrix mineralization. Conversely, silencing Creld2 expression inhibits BMP9-induced osteogenic differentiation. Exogenous Creld2 augments BMP9-induced ectopic bone formation and matrix mineralization, whereas silencing Creld2 expression significantly diminishes BMP9-induced bone formation and matrix mineralization. We further demonstrate that Creld2 is located in ER, and ER stress inducers can effectively potentiate BMP9-induced ALP activity. Thus, our results strongly suggest that Creld2 may be directly regulated by BMP9 via Smad signaling pathway and the ER stress may play an important role in regulating BMP9-induced osteogenic differentiation in MSCs.

While it remains to be thoroughly investigated if BMP9 plays any significant roles in skeletal development, recent studies indicate that aberrant BMP9 osteogenic activity may be associated with certain clinical disorders. BMP9 was shown to cause heterotopic ossification in injured muscle, which could be significantly blocked by the soluble form of BMP9 receptor ALK1 in a mouse model [59]. These findings suggest that BMP9 may be considered a candidate for involvement in heterotopic ossification physiopathology with its activity depending on the skeletal muscle microenvironment. More recently, it has been reported that the severity of ossification of the posterior longitudinal ligament (OPLL) may be associated with genetic variations in a 3-kb BMP9 locus in a Chinese population [60]. Analysis of the complete BMP9 gene on single markers and haplotypes in 450 patients with OPLL and in 550 matched controls and subsequent linkage disequilibrium (LD) analysis identified one 3-kb block of intense LD in BMP9 and one specific haplotype CTCA, which should contain the OPLL-associated risk alleles and be considered as a risk factor for OPLL [60]. Thus, future directions should be directed at elucidating the in vivo functions of BMP9 during development and in adult skeletal homeostasis.

Creld2 was originally identified as a novel endoplasmic reticulum stress-inducible protein localized in the ER-Golgi apparatus [31], [32], its biological functions are largely undefined, especially in the context of osteotgenic differentiation of MSCs. CRELD2 is the second member of the CRELD family of proteins [31], [32], [61]. CRELD2 gene is conserved in chimpanzee, Rhesus monkey, dog, cow, mouse, rat, chicken, zebrafish, fruit fly, mosquito, and C.elegans. The only other CRELD family member CRELD1 (aka, AVSD2) has mutations in CRELD1 and is associated with cardiac atrioventricular septal defects (AVSD). CRELD2 is ubiquitously expressed during development and in mature tissues, with the highest levels in adult endocrine tissues [31]. Recently, a specific CRELD2 isoform (CRELD2β) was implicated as a regulator of α4β2 nicotinic acetylcholine receptor expression [62]. Our gene expression profiling analysis indicates that Creld2 is among the top up-regulated genes in BMP9-stimulated MSCs [29]. Here, we confirm that Creld2 is a direct downstream target of BMP9/Smad signaling pathway and plays an important role of BMP9-induced terminal osteogenic differentiation, although detailed mechanism underlying Creld2’s role in BMP9-mediated osteogenic signaling remains to be thoroughly investigated.

Several recent studies strongly suggest that ER stress may play an important role in bone formation. An ER stress transducer and a member of the CREB/ATF family, OASIS, was induced by BMP2 and shown to play an important role in bone formation and fracture healing [33]–[35]. Another ER stress sensor PERK was shown to participate in BMP2-induced during osteoblast differentiation and to activate the PERK-eIF2α-ATF4 signaling pathway followed by the promotion of gene expression essential for osteogenesis [36], [37]. The C/EBP family member CHOP is a multifunctional ER-induced transcription factor and also involved in regulating bone formation [38]–[40]. It has been reported that the Site-1 protease (S1P) is necessary for a specialized ER stress response required for endochondral ossification and growth plate development [41], [42]. Furthermore, we have recently demonstrated that MAPK signaling pathway is involved in regulating BMP9-induced osteogenic differentiation [43], [44]. Thus, it would be interesting to further elucidate how Creld2 regulates BMP9-induced osteogenesis during ER stress.

In summary, we find that Creld2 is up-regulated in MSCs by BMP9 via the Smad signaling pathway. Exogenous expression of Creld2 in MSCs potentiates BMP9-induced osteogenic markers and matrix mineralization in vitro. Conversely, silencing Creld2 expression diminishes BMP9-induced osteogenic differentiation. Exogenous Creld2 augments BMP9-induced ectopic bone formation and mature mineralization, whereas silencing Creld2 expression significantly diminishes BMP9-induced bone formation and matrix mineralization. Creld2 is located on ER and ER stress inducers potentiate BMP9-induced osteogenic differentiation. Our results strongly suggest that ER stress factor Creld2 may be directly regulated by BMP9 and play an important role in regulating terminal osteogenic differentiation in BMP9-stimulated MSCs. Future studies should be directed at least in part to the understanding of molecular mechanisms underlying Creld2’s role in BMP9-induced osteogenesis.

## Supporting Information

Table S1 **Top 20 BMP9 up-regulated genes in MSCs.**
(XLS)

Table S2 **Primers used for sqPCR, ChIP, siRNA and cloning.**
(XLS)
